# Transarterial Chemoembolization Combined With Endoscopic Therapy Is Beneficial for Unresectable Hepatocellular Carcinoma With Esophagogastric Varices

**DOI:** 10.3389/fonc.2021.783574

**Published:** 2021-12-02

**Authors:** Ziwen Tao, Yuying Ruan, Zhi Peng, Kai Zhang, Yanjing Gao

**Affiliations:** ^1^ Department of Gastroenterology, Qilu Hospital, Cheeloo College of Medicine, Shandong University, Jinan, China; ^2^ Department of Interventional Radiography, Qilu Hospital, Cheeloo College of Medicine, Shandong University, Jinan, China

**Keywords:** hepatocellular carcinoma, esophagogastric varices, transarterial chemoembolization, endoscopic variceal ligation, endoscopic injection sclerotherapy

## Abstract

**Background:**

The efficacy of transarterial chemoembolization (TACE) combined with endoscopic therapy for unresectable hepatocellular carcinoma with esophagogastric varices remains unclear.

**Methods:**

The study has been registered on ClinicalTrials.gov with the number NCT05017922 (https://register.clinicaltrials.gov). Eligible patients were divided into combined group (received TACE plus endoscopic therapy) and control group (only received TACE). The occurrence of death and bleeding episodes during the follow-up was recorded. Kaplan–Meier analysis was used to compare outcomes between the two groups. Cox proportional hazard model was used to determine independent predictors for the survival.

**Results:**

Eighty-nine patients were included, 42 in the combined group, others in the control group. During the follow-up, 51 patients died, the 1-year, 2-year, and 3-year survival rates were 64.9%, 45.5%, and 34.5%. The cumulative survival was significantly higher in the combined group than in the control group (*p* = 0.027); the 1-year, 2-year, and 3-year survival rates were 75.5%, 55.9%, 43.8% and 55.0%, 35.9%, 26.6%, respectively. Forty-four patients experienced bleeding, the bleeding rate was significantly higher in the control group than in the combined group (77.4% vs. 56.8%, *p* = 0.016). Multivariate analysis showed that treatment, hemoglobin, portal vein tumor thrombosis, and aspartate aminotransferase were independent predictors for overall survival; the first three factors were also independent predictors for bleeding-free survival. Patients who received primary prophylaxis had longer overall survival (*p* = 0.042) and bleeding-free survival (*p* = 0.029) than those who received secondary prophylaxis.

**Conclusions:**

TACE combined with endoscopic therapy significantly improved survival and reduced bleeding rates in unresectable hepatocellular carcinoma with esophagogastric varices patients. Portal vein tumor thrombosis was a strong negative prognostic factor for both overall survival and bleeding-free survival. Primary prophylaxis improved survival benefits compared with secondary prophylaxis.

## 1 Introduction

Liver cancer is the sixth most common malignancy and the third leading cancer-related mortality in the world (1). Due to the occult characteristics of the occurrence, about 70% of patients are diagnosed at an advanced stage and lose opportunities for surgical treatment (2). Transarterial chemoembolization (TACE) plays an important role as first-line therapy for unresectable hepatocellular carcinoma (HCC) and has been reported that it could improve 2-year survival when compared with conservative management for patients with unresectable HCC (OR = 0.53, 95% CI: 0.32–0.89, *p* = 0.017) (3). In addition, drug-eluting bead TACE is also considered as an effective down-staging treatment for unresectable HCC, with a down-staging success rate of 59.4%. In subsequent radical treatment, the complete response rate was 81.3% (4).

Liver cirrhosis results from a variety of causes can lead to HCC. An analysis showed that hepatitis B or hepatitis C is responsible for about 76% of the global incidence of the HCC (5). Portal hypertension is an important component in the natural history of liver cirrhosis, multiple portosystemic collaterals formatted when hepatic venous pressure gradient≥10 mmHg (6). Esophagogastric varices (EGV) is the most common form, which occurs in about 50% of liver cirrhosis patients. Since variceal hemorrhage is the major cause of death in patients with liver cirrhosis, with a 6-week mortality rate as high as 15%–20%, close monitoring and treatment are essential (7, 8). Endoscopic therapy, including endoscopic variceal ligation (EVL) and endoscopic injection sclerotherapy (EIS), is often used to provide primary and secondary prophylaxis of EGV and is considered to be the first-line treatment of acute esophagogastric variceal bleeding (EGVB) (9).

A propensity score matching study found that HCC patients with EGV had poorer liver functional reserve and that EGV was an independent risk factor for poor prognosis after TACE (10). Kim et al. (11) also demonstrated that in HCC patients, the occurrence of variceal bleeding could increase the risk of mortality (HR = 1.39, 95% CI: 1.06–1.82, *p* = 0.015), whereas primary prophylaxis of EGV could significantly reduce it (HR = 0.54, 95% CI: 0.33–0.88, *p* = 0.014). Emergency EVL has also been confirmed to be a safe and effective treatment for acute variceal hemorrhage in patients with HCC associated with portal vein tumor thrombosis (PVTT) (12). Since the efficacy of TACE combined with endoscopic therapy for unresectable HCC complicated with EGV remains unclear, we want to explore it and seek out predictors associated with survival through this non-randomized concurrent controlled trial to provide some reference for the treatment of such patients in the future.

## 2 Materials and Methods

### 2.1 Patients and Groups

All the eligible HCC patients treated in Qilu Hospital of Shandong University from 2017 to 2020 were evaluated prospectively and consecutively, who met the following inclusion criteria: (1) HCC was diagnosed in accordance with the 2017 edition of diagnosis guidelines (13), and all patients were in CNLC stage Ib to IIIa, and treated with TACE; (2) EGV was demonstrated through endoscopic examination; (3) Child-Pugh grade A or B, or grade C patients improved liver function to grade A or B through aggressive treatment; and (4) age between 18 and 75 years. The exclusion criteria were the following: (1) HCC with diffuse or distant metastasis, or with other systemic malignancies; (2) severe jaundice, hepatic encephalopathy, refractory ascites, or hepatorenal syndrome; (3) severe cardiac, cerebrovascular, lung, and renal diseases and cannot tolerate endoscopic treatment; (4) severe coagulation dysfunction; (5) severe infection, bleeding with unstable vital signs; (6) history of liver surgery; and (7) cannot or refuse to sign the informed consent.

Eligible patients were divided into two groups: combined group (received TACE plus endoscopic therapy) and control group (only received TACE); the treatment was decided by patients after they were informed of the risks and uncertain benefits of endoscopic therapy.

### 2.2 Study Design and Data Collection

Eligible patients were followed up and data were prospectively collected after receiving the standard treatment regimens to evaluate the efficacy of the therapy. Informed consents were obtained from all respondents, and the ethics of this study was approved by the Ethics Committee of Scientific Research of Qilu Hospital of Shandong University (No. 2016009). The study has been registered on ClinicalTrials.gov with the number NCT05017922.

The following data were collected: age, gender, etiology of the underlying liver disease, presence or absence of ascites, history of EGVB, alanine aminotransferase, aspartate aminotransferase (AST), total bilirubin, albumin, creatinine, hemoglobin (Hb), platelet, prothrombin time, alpha-fetoprotein, tumor number, tumor size, PVTT, Child-Pugh score, and BCLC stage.

### 2.3 Treatment

#### 2.3.1 TACE

The 4-French catheter was inserted from the right femoral artery using the Seldinger technique, and the supplying artery of HCC was evaluated by visceral angiography. Then, the tip of the catheter was advanced into the left or right hepatic artery or tumor-feeding artery based on the tumor location and size. 5-Fu was injected first after safe fixed of the catheter. Then, the chemolipiodolization was performed, using oxaliplatin, epirubicin, and lipiodol. If stagnant flow did not show in the chemolipiodolization arterial territory, pure lipiodol was then injected. If the tumor-feeding artery still cannot be completely embolized, several absorbable gelatin sponge particles would be injected. The whole procedure was performed under fluoroscopy. This treatment regimen was conducted consistently in this study, regardless of tumor size and number.

#### 2.3.2 Endoscopic Therapy

Symptomatic supportive treatment was given preoperatively to reduce portal venous pressure and correct anemia in all patients and ensure that vital signs were stable. After the site of esophageal varices was identified by the gastroscopy, spiral ligation using the EVL device (COOK, MBL-6-F) was performed and ensure the varices were fully inhaled, one to three rubber bands were used totally. After EVL, water was sprayed to the ligation sites to check for bleeding. If gastric varices were found, EIS was performed using the “sandwich method” of hypertonic glucose-tissue adhesive-normal saline. Make sure the needle entered the varicose vein before injecting drugs, and repeated the injection if necessary. When the EIS is accomplished, press on the puncture site to stop bleeding and then observe the sclerosis of varicose veins.

### 2.4 Follow-Up and Study Endpoints

After the first TACE, computerized tomography, and/or magnetic resonance imaging, tumor markers, liver function, and blood routine test were reexamined every 4–6 weeks. Subsequent TACE was determined according to the follow-up results. About two to four cycles of TACE were required for large HCC. Adjuvant radiofrequency ablation was given for all the small lesions found surrounding the primary lesions. Patients who received endoscopic therapy were reexamined 1–2 weeks after the first therapy, and subsequent endoscopic therapy was performed according to the varicose veins, until varicose veins disappeared or basically disappeared. After all the treatment was done, death and bleeding episodes were assessed every 3 months by phone call or outpatient service, then changed to every 6 months after a year of follow-up. It stopped in July 2020 or the day of death or the day of loss to follow-up or follow-up for 3 years.

### 2.5 Statistical Analysis

Data analysis was performed through the statistical software IBM SPSS Statistical 26.0 (SPSS Inc, Chicago, Illinois, USA). Continuous variables were expressed as the mean ± SD, whereas categorical variables were expressed as numbers. Continuous variables were compared using the Wilcoxon rank-sum test or Student’s *t*-test, and categorical variables were compared using the Fisher exact test or Chi-square test. Kaplan–Meier analysis was used to compare cumulative survival and bleeding episodes between the two groups. Cox proportional hazard regression model was used to determine independent risk factors for the overall survival (OS) and bleeding-free survival. Statistical significance was considered when a two-tailed *p* < 0.05.

## 3 Results

### 3.1 Patient Characteristics

A total of 89 patients diagnosed with unresectable HCC complicated with EGV were included in this study during 2017–2020. The median age was 59 years (range from 32 to 78 years), 76 were males and 13 were females. The characteristics of the included patients are listed in **Table 1**. In the combined group (*n* = 42), 88.1% were males, and the mean age and Child-Pugh score were 58.4 ± 7.6 years and 6.9 ± 1.6, respectively. The etiologies of underlying liver disease were hepatitis B (*n* = 35, 83.3%), hepatitis C (*n* = 2, 4.8%), and alcoholic hepatitis (*n* = 5, 11.9%). Twenty-four (57.1%) developed ascites, 24 (57.1%) had a history of EGVB, and 10 (23.8%) had PVTT. Twenty-one of them were Child-Pugh grade A, 18 were grade B, and 3 improved from grade C to grade B. In the control group (*n* = 47), 83.0% were males, and the mean age and Child-Pugh score were 58.0 ± 9.7 years and 6.3 ± 1.5, respectively. The etiologies of underlying liver disease were hepatitis B (*n* = 43, 91.5%), hepatitis C (*n* = 1, 2.1%), and alcoholic hepatitis (*n* = 3, 6.4%). Eighteen (38.3%) developed ascites, 23 (48.9%) had a history of EGVB, and 15 (31.9%) had PVTT. Twenty-nine of them were Child-Pugh grade A, 16 were grade B, and 2 improved from grade C to grade B. None of the included patients have received systemic chemotherapy or immunotherapy during the follow-up period. No significant difference in these characteristics was found.

**Table 1:** Baseline characteristics of all patients in the two groups.

Characteristics	Combined group (*n* = 42)	Compared group (*n* = 47)	*p*-value
Age (years)	58.4 ± 7.6	58.0 ± 9.7	0.838
Sex (male/female)	37/5	39/8	0.495
Etiology (*n*)			
Hepatitis B	35	43	0.517
Hepatitis C	2	1	
Alcoholic Hepatitis	5	3	
Ascites (*n*)	24	18	0.164
EGVB (*n*)	24	23	0.439
ALT (U/L)	41.4 ± 36.3	49.0 ± 34.9	0.318
AST (U/L)	51.9 ± 33.3	63.1 ± 43.2	0.180
TBIL (μmol/L)	24.8 ± 12.2	32.1 ± 43.3	0.295
Alb (g/L)	37.0 ± 5.3	36.7 ± 6.7	0.783
Cr (mg/dl)	69.3 ± 14.8	65.9 ± 14.0	0.263
Hb (g/L)	111.4 ± 30.9	116.3 ± 28.6	0.443
PLT (×10^9^/L)	96.1 ± 69.2	100.3 ± 53.5	0.751
PT (s)	14.4 ± 2.1	13.7 ± 2.0	0.110
AFP (ng/ml)	1,703.0 ± 5,357.2	3,055.5 ± 7,141.0	0.335
Tumor number (*n*)			
1	18	25	0.330
≥2	24	22	
Tumor size (*n*)			
<5 cm	36	33	0.080
≥5 cm	6	14	
PVTT (*n*)	10	15	0.396
Child-Pugh score	6.9 ± 1.6	6.3 ± 1.5	0.121
Child-Pugh grade (*n*)			
A	21	29	0.567
B	18	16	
C	3	2	
BCLC stage (*n*)			
A2	7	10	0.337
A3	9	15	
A4	0	1	
B	26	21	

Values are expressed as mean ± SD. Abbreviations: EGVB, esophagogastric varices bleeding; ALT, alanine aminotransferase; AST, aspartate aminotransferase; TBIL, total bilirubin; Alb, albumin; Cr, creatinine; Hb, hemoglobin; PLT, platelet; PT, prothrombin time; AFP, alpha fetoprotein; PVTT, portal vein tumor thrombosis; BCLC, Barcelona Clinic Liver Cancer.

### 3.2 Overall Survival

**Figure 1:**
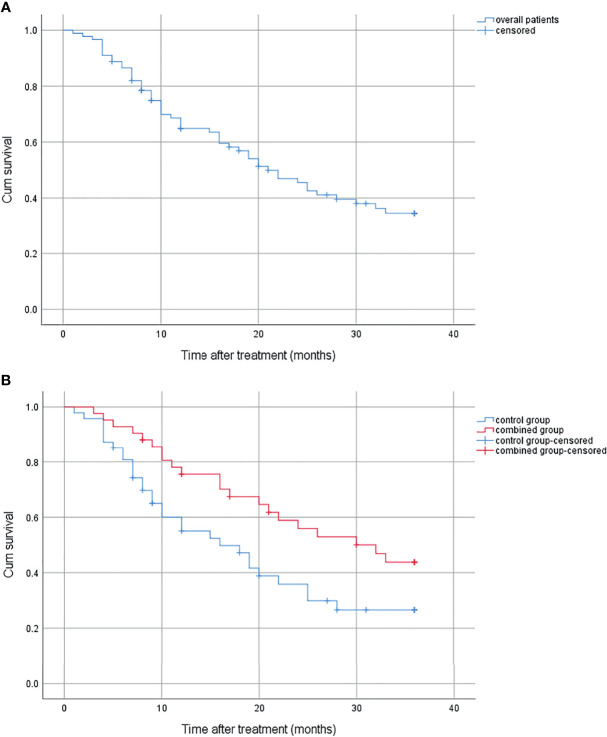
**(A)** Cumulative survival for overall patients. **(B)** Cumulative survival for the combined group and the control group.

There were 51 patients who died during the follow-up, 21 in the combined group and 30 in the control group; 9 (42.9%) patients died from bleeding in the combined group, and 17 (56.7%) in the control group. The survival period of the whole group was 1–36 months (median, 21 months); the 1-year, 2-year, and 3-year survival rates were 64.9%, 45.5%, and 34.5% (**Figure 1A**). The cumulative survival was significantly higher in the combined group than in the control group (*p* = 0.027), with the median survival being 32 and 16 months, and the 1-year, 2-year, and 3-year survival rates were 75.5%, 55.9%, 43.8% and 55.0%, 35.9%, 26.6%, respectively (**Figure 1B**).

### 3.3 Bleeding Episodes

**Figure 2:**
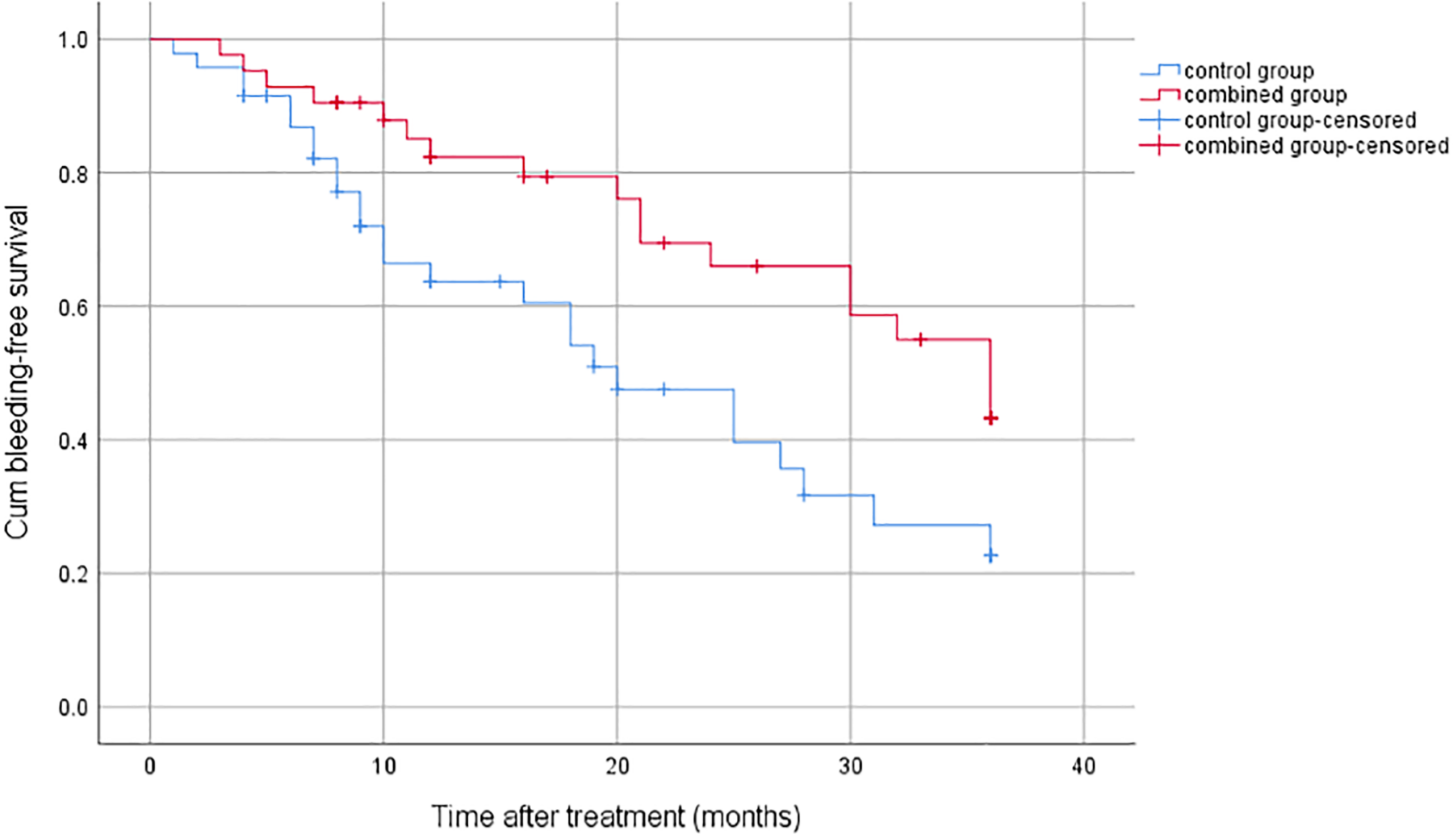
Cumulative bleeding-free survival for the combined group and the control group.

There were 44 patients who experienced bleeding during the follow-up, 18 in the combined group and 26 in the control group. The bleeding rate was significantly higher in the control group than in the combined group (77.4% vs. 56.8%, *p* = 0.016) (**Figure 2**).

### 3.4 Predictors of OS

All the baseline data of the patients were analyzed through univariate and multivariate Cox proportional hazard regression model to explore possible predictors of OS. The univariate analysis showed that treatment (HR = 0.539, 95% CI: 0.307–0.946, *p* = 0.031), Child-Pugh score (HR = 1.227, 95% CI: 1.035–1.454, *p* = 0.019), Hb (HR = 0.990, 95% CI: 0.981–0.999, *p* = 0.030), AST (HR = 1.010, 95% CI: 1.004–1.015, *p* = 0.001), alanine aminotransferase (HR = 1.009, 95% CI: 1.002–1.017, *p* = 0.011), albumin (HR = 0.945, 95% CI: 0.903–0.989, *p* = 0.015), total bilirubin (HR = 1.011, 95% CI: 1.004–1.017, *p* = 0.001), and PVTT (HR = 3.913, 95% CI: 2.163–7.078, *p* < 0.01) were risk factors for OS. The multivariate analysis showed that treatment (HR = 0.520, 95% CI: 0.277–0.977, *p* = 0.042), Hb (HR = 0.985, 95% CI: 0.974–0.996, *p* = 0.006), AST (HR = 1.010, 95% CI: 1.001–1.020, *p* = 0.033), and PVTT (HR = 4.441, 95% CI: 2.336–8.440, *p* < 0.01) were independent prognostic factors for OS (**Table 2**).

**Table 2:** Univariate and multivariate analysis of predictors for OS.

Variables	Univariate analysis		Multivariate analysis		
	HR	*p*-value	HR	95% CI	*p*-value
Age (years)	1.012	0.489			
Sex (male/female)	1.194	0.663			
Etiology	1.551	0.352			
Ascites	1.110	0.710			
EGVB	1.552	0.126			
ALT (U/L)	1.009	0.011	0.995	0.984–1.006	0.364
AST (U/L)	1.010	0.001	1.010	1.001–1.020	0.033
TBIL (μmol/L)	1.011	0.001	1.004	0.995–1.012	0.390
Alb (g/L)	0.945	0.015	0.989	0.924–1.058	0.739
Cr (mg/dl)	0.998	0.824			
Hb (g/L)	0.990	0.030	0.985	0.974–0.996	0.006
PLT (×10^9^/L)	1.002	0.347			
PT (s)	1.113	0.136			
AFP (ng/ml)	1.000	0.005			
Tumor number	1.085	0.772			
Tumor size (cm)	1.301	0.441			
PVTT	3.913	<0.01	4.441	2.336–8.440	<0.01
Child-Pugh score	1.227	0.019	1.075	0.806–1.435	0.622
BCLC stage	1.173	0.573			
Treatment	0.539	0.031	0.520	0.277–0.977	0.042

OS, overall survival; HR, hazard ratio; CI, confidence interval; EGVB, esophagogastric varices bleeding; ALT, alanine aminotransferase; AST, aspartate aminotransferase; TBIL, total bilirubin; Alb, albumin; Cr, creatinine; Hb, hemoglobin; PLT, platelet; PT, prothrombin time; AFP, alpha fetoprotein; PVTT, portal vein tumor thrombosis; BCLC, Barcelona Clinic Liver Cancer.

### 3.5 Predictors of Bleeding-Free Survival

All the baseline data of the patients were analyzed through univariate and multivariate Cox proportional hazard regression model to explore possible predictors of OS. The univariate analysis showed that treatment (HR = 0.487, 95% CI: 0.265–0.894, *p* = 0.020), Child-Pugh score (HR = 1.224, 95% CI: 1.020–1.470, *p* = 0.030), Hb (HR = 0.983, 95% CI: 0.974–0.993, *p* = 0.001), AST (HR = 1.007, 95% CI: 1.001–1.014, *p* = 0.024), total bilirubin (HR = 1.010, 95% CI: 1.001–1.018, *p* = 0.022), PVTT (HR = 4.071, 95% CI: 2.045–8.104, *p* < 0.01), and history of EGVB (HR = 2.315, 95% CI: 1.223–4.382, *p* = 0.010) were risk factors for bleeding-free survival. The multivariate analysis showed that treatment (HR = 0.384, 95% CI: 0.190–0.773, *p* = 0.007), Hb (HR = 0.973, 95% CI: 0.955–0.991, *p* = 0.003), and PVTT (HR = 4.829, 95% CI: 2.231–10.452, *p* < 0.01) were independent prognostic factors for bleeding-free survival (**Table 3**).

**Table 3:** Univariate and multivariate analysis of predictors for bleeding-free survival.

Variables	Univariate analysis		Multivariate analysis		
	HR	*p-*value	HR	95% CI	*p-*value
Age (years)	1.027	0.167			
Sex (male/female)	1.174	0.716			
Etiology	1.712	0.306			
Ascites	1.160	0.623			
EGVB	2.315	0.010	0.796	0.275–2.308	0.674
ALT (U/L)	1.007	0.128			
AST (U/L)	1.007	0.024	1.004	0.996–1.011	0.310
TBIL (μmol/L)	1.010	0.022	1.005	0.996–1.014	0.299
Alb (g/L)	0.952	0.058			
Cr (mg/dl)	1.000	0.992			
Hb (g/L)	0.983	0.001	0.973	0.955–0.991	0.003
PLT (×10^9^/L)	1.002	0.544			
PT (s)	1.091	0.277			
AFP (ng/ml)	1.000	0.008			
Tumor number	0.962	0.897			
Tumor size (cm)	1.403	0.347			
PVTT	4.071	<0.01	4.829	2.231–10.452	<0.01
Child-Pugh score	1.224	0.030	1.034	0.822–1.301	0.777
BCLC stage	1.075	0.811			
Treatment	0.487	0.020	0.384	0.190–0.773	0.007

HR, hazard ratio; CI, confidence interval; EGVB, esophagogastric varices bleeding; ALT, alanine aminotransferase; AST, aspartate aminotransferase; TBIL, total bilirubin; Alb, albumin; Cr, creatinine; Hb, hemoglobin; PLT, platelet; PT, prothrombin time; AFP, alpha fetoprotein; PVTT, portal vein tumor thrombosis; BCLC, Barcelona Clinic Liver Cancer.

### 3.6 Effects of PVTT on OS and Bleeding Episodes

According to the correlation analysis, we found that the occurrence of PVTT was significantly negatively correlated with OS and bleeding-free survival. Based on this finding, we respectively compared OS and bleeding episodes with or without PVTT.

**Figure 3:**
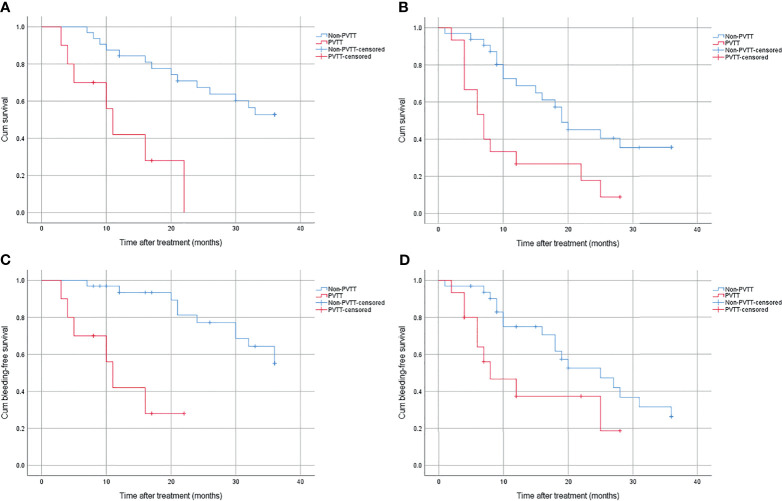
**(A)** Cumulative survival for patients with or without PVTT in the combined group. **(B)** Cumulative survival for patients with or without PVTT in the control group. **(C)** Cumulative bleeding-free survival for patients with or without PVTT in the combined group. **(D)** Cumulative bleeding-free survival for patients with or without PVTT in the control group.

No matter what kind of treatment was conducted, patients with PVTT had worse OS than those without. In the combined group, the 1-year, 2-year, and 3-year survival rate were 42.0%, 0%, 0% and 84.4%, 67.3%, 52.7% for patients with and without PVTT (*p* < 0.01), respectively (**Figure 3A**). In the control group, the 1-year, 2-year, and 3-year survival rate were 26.7%, 17.8%, 0% and 68.7%, 45.0%, 35.4% for patients with and without PVTT (*p* = 0.003), respectively (**Figure 3B**).

Patients with PVTT also had worse bleeding-free survival than those without, regardless of treatment method. In the combined group, the bleeding rate was 72% and 49.5% for patients with and without PVTT (*p* < 0.01), respectively (**Figure 3C**). In the control group, the bleeding rate was 81.3% and 73.7% for patients with and without PVTT (*p* = 0.033), respectively (**Figure 3D**).

### 3.7 Effects of Primary Prophylaxis on OS and Bleeding Episodes

**Figure 4:**
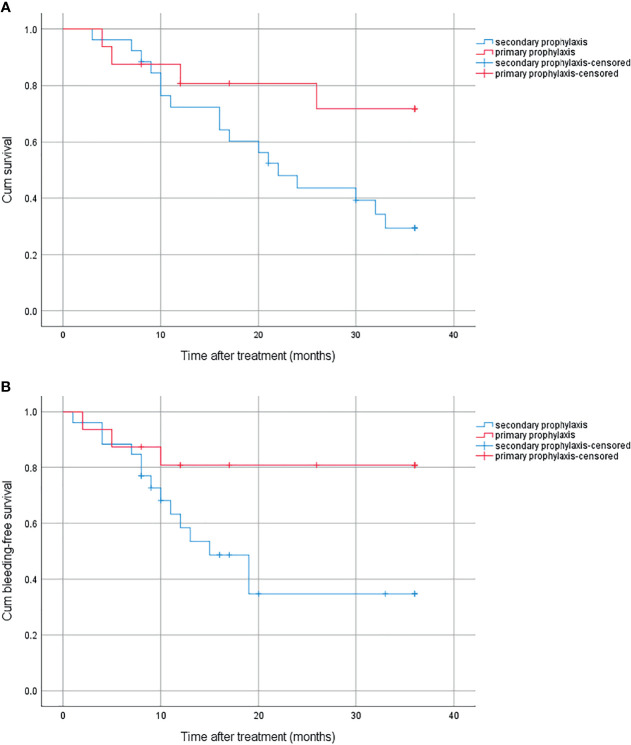
**(A)** Cumulative survival for primary prophylaxis and secondary prophylaxis. **(B)** Cumulative bleeding-free survival for primary prophylaxis and secondary prophylaxis.

There were 16 patients who received primary prophylaxis in the combined group. The cumulative survival was significantly higher in patients who received primary prophylaxis than those who received secondary prophylaxis, with 1-year, 2-year, and 3-year survival rates of 80.8%, 80.8%, 71.8% and 72.4%, 43.6%, 29.4% (*p* = 0.042), respectively (**Figure 4A**). The bleeding rate was significantly higher in the latter (19.2% vs. 65.3%, *p* = 0.029) (**Figure 4B**).

## 4 Discussion

This is the first prospective study to demonstrate that TACE combined with endoscopic therapy significantly improved survival and reduced bleeding rates in patients with unresectable HCC complicated with EGV. We observed that TACE combined with endoscopic therapy yielded survival outcomes significantly superior to TACE alone for unresectable HCC complicated with EGV, with 1-year, 2-year, and 3-year survival rates of 75.5%, 55.9%, 43.8% and 55.0%, 35.9%, 26.6% (*p* = 0.027), and the bleeding rate was 56.8% and 77.4% (*p* = 0.016), respectively. Treatment, Hb, PVTT, and AST were independent prognostic factors for OS; the first three factors were also independent prognostic factors for bleeding-free survival. No matter what kind of treatment was conducted, patients with PVTT had worse OS and bleeding-free survival than those without. Patients who received primary prophylaxis had longer overall survival (*p* = 0.042) and bleeding-free survival (*p* = 0.029) than those who received secondary prophylaxis.

Studies have shown that the in-hospital mortality rate of HCC patients with variceal hemorrhage was as high as 20.5%, and their OS was worse than those without EGVB (median, 3.5 vs. 7.5 months, *p* < 0.001), indicating that the occurrence of EGV significantly affects the survival of HCC, and it has a great possibility to induce death by causing bleeding before the HCC progresses (14). Therefore, the prevention of variceal bleeding is very necessary to improve the survival of HCC. Endoscopic therapy has been widely used in the primary and secondary prophylaxis of EGV. Our results showed that endoscopic therapy combined with TACE could significantly improve outcomes for unresectable HCC patients complicated with EGV, especially in patients who received primary prophylaxis, which is consistent with previous studies (15). Chen et al. (16) performed secondary prophylactic endoscopic therapy for EGVB on 192 HCC patients and found that it could provide survival benefits for these patients whether through EVL or EIS, with 6-month, 1-year, and 5-year cumulative rebleeding rates of 40.9%, 49.3%, and 71.2%, and cumulative mortality rates of 33.5%, 45.8%, and 65.7%, respectively. Kim et al. (11) affirmed the effectiveness of primary prophylaxis in HCC patients, with 1- and 3-year cumulative variceal bleeding rates of 20.4% and 30.4%, respectively. This suggested that primary prophylaxis was superior to secondary prophylaxis in reducing the risk of rebleeding.

We also found that, in addition to treatment method, levels of Hb and AST, and PVTT status could influence outcomes of patients. These have also been demonstrated in previous studies. Low levels of Hb were considered to be an independent risk factor of rebleeding after EVL treatment in liver cirrhosis patients with esophageal varices (OR = 17.3491, 95% CI: 4.00–75.34, *p* = 0.005) (17). The occurrence of early ascites after drug-eluting bead TACE in HCC patients was associated with poor prognosis (median OS, 17 months), which was revealed to be influenced by levels of Hb before treatment (18). In patients with spontaneous ruptured HCC who were treated with TACE, higher Hb was independently associated with 30-day survival (OR = 0.609, *p* = 0.036) (19). Higher Hb after TACE combined with external beam radiotherapy for unresectable HCC was a predictor of successful treatment (*p* = 0.016) (20).

As an indicator of liver function, AST represents the degree of liver damage and the reserve of liver function. Liver injury from any causes can lead to elevated AST in the blood. In HCC patients who have undergone radical hepatectomy, AST could be used to predict early postoperative recurrence and post-recurrence survival (21). Low levels of alanine aminotransferase-to-AST ratio was associated with longer survival in primary HCC patients (*p* < 0.05) (22).

PVTT is the most common form of macrovascular invasion of HCC, which is found in approximately 10%–60% patients at the time of diagnosis of HCC (23, 24). It is a strong negative prognostic factor, and once it is present, HCC is classified into advanced stage, with an OS ranging from 2 to 4 months if only treated with conservative treatment (25). Portal vein pressure increased due to the PVTT and then numerous collateral veins around the obstructed portal vein formed, which may lead to the development or aggravation of EGV and increase the potential bleeding complications (26). Lim et al. (27) demonstrated that patients with PVTT had a higher proportion of high-risk varices (23.0 vs. 13.3%, *p* = 0.003) and cumulative variceal bleeding rate (4.5 vs. 0.4% at 1 year, *p* = 0.009) than those without, and Vp4 PVTT was an independent predictor for high-risk varices (aOR = 3.345, 95% CI: 1.457–7.680, *p* < 0.05). TACE may provide survival benefits for HCC patients with PVTT, and can be safely performed as long as liver function is good and collateral circulation around the embolization site is abundant (28). Niu et al. (29) found that TACE could significantly improve survival compared with conservative treatment for HCC with any type of PVTT (OS, 8.67 vs. 1.4 months, *p* < 0.001), and the extent of PVTT was independent prognostic factors (OR = 1.856, 95% CI: 1.449-2.377, *p* < 0.001). Similar conclusions were also confirmed by Luo et al. (30).

There are some limitations in our study. First, the non-randomized design of this study introduces a potential selected bias that needs randomized trial to reduce it. Second, the single-center study with a small sample size and relatively short study period, and HBV infection as the main etiology, entails that the extrapolation of the conclusion needs to be further verified. Third, adjuvant radiofrequency ablation was given for all the small lesions found surrounding the primary lesions; whether it has an influence on the survival benefit still needs further verification.

## 5 Conclusion

We observed that TACE combined with endoscopic therapy significantly improved survival and reduced bleeding rates in patients with unresectable HCC complicated with EGV. PVTT was a strong negative prognostic factor for both OS and bleeding-free survival. Primary prophylaxis improved survival benefits compared with secondary prophylaxis. Multicenter prospective randomized control trials are still needed to verify the accuracy of the conclusions in the future.

## Data Availability Statement

The raw data supporting the conclusions of this article will be made available by the authors, without undue reservation.

## Ethics Statement

The studies involving human participants were reviewed and approved by the Medical Ethics Committee of the Qilu Hospital of Shandong University. The patients/participants provided their written informed consent to participate in this study.

## Author Contributions

ZT, YR, and ZP contributed to conception and design of the study. YR and ZP collected the data. ZT and YR performed the statistical analysis. ZT wrote the first draft of the manuscript. All authors contributed to manuscript revision, read, and approved the submitted version.

## Funding

This study was supported by a grant from the Natural Science Foundation of Shandong Province (No. ZR2019MH112).

## Conflict of Interest

The authors declare that the research was conducted in the absence of any commercial or financial relationships that could be construed as a potential conflict of interest.

## Publisher’s Note

All claims expressed in this article are solely those of the authors and do not necessarily represent those of their affiliated organizations, or those of the publisher, the editors and the reviewers. Any product that may be evaluated in this article, or claim that may be made by its manufacturer, is not guaranteed or endorsed by the publisher.

## Abbreviations

HCC, hepatocellular carcinoma; TACE, transarterial chemoembolization; EGV, esophagogastric varices; EVL, endoscopic variceal ligation; EIS, endoscopic injection sclerotherapy; EGVB, esophagogastric varices bleeding; PVTT, portal vein tumor thrombosis; AST, aspartate aminotransferase; Hb, hemoglobin; OS, overall survival.
